# First record of growth patterns in a Cambrian annelid

**DOI:** 10.1098/rsos.221400

**Published:** 2023-04-26

**Authors:** Hatena Osawa, Jean-Bernard Caron, Robert R. Gaines

**Affiliations:** ^1^ Department of Ecology and Evolutionary Biology, University of Toronto, 25 Willcocks Street, Toronto, Ontario, Canada M5S 3B2; ^2^ Department of Natural History, Royal Ontario Museum, 100 Queen's Park, Toronto, Ontario, Canada M5S 2C6; ^3^ Department of Earth Sciences, University of Toronto, 22 Ursula Franklin Street, Toronto, Ontario, Canada M5S 3B1; ^4^ Geology Department, Pomona College, 185 E Sixth Street, Claremont, CA 91711, USA

**Keywords:** Burgess Shale, stem-group annelid, polychaete, gregarious behaviour, body tagmatization, development

## Abstract

Early annelid evolution is mostly known from 13 described species from Cambrian Burgess Shale-type Lagerstätten. We introduce a new exceptionally well-preserved polychaete, *Ursactis comosa* gen. et sp. nov., from the Burgess Shale (Wuliuan Stage). This small species (3–15 mm) is the most abundant Cambrian polychaete known to date. Most specimens come from Tokumm Creek, a new Burgess Shale locality in northern Kootenay National Park, British Columbia, Canada. *Ursactis* has a pair of large palps, thin peristomial neurochaetae and biramous parapodia bearing similarly sized capillary neurochaetae and notochaetae, except for segments six to nine, which also have longer notochaetae. The number of segments in this polychaete range between 8 and 10 with larger individuals having 10 segments. This number of segments in *Ursactis* is remarkably small compared with other polychaetes, including modern forms. Specimens with 10 segments show significant size variations, and the length of each segment increases with the body length, indicating that body growth was primarily achieved by increasing the size of existing segments rather than adding new ones. This contrasts with most modern polychaetes, which typically have a larger number of segments through additions of segments throughout life. The inferred growth pattern in *Ursactis* suggests that annelids had evolved control over segment addition by the mid-Cambrian.

## 1. Introduction

Annelids are a morphologically and ecologically diverse group that includes terrestrial species, such as earthworms and leeches, aquatic polychaetes and abyssal tubeworms. Annelids evolved during the Cambrian Explosion, and their earliest diversity is preserved in several Burgess Shale-type deposits [1–9]. Thirteen Cambrian polychaete species have been described so far from these deposits, six of them from the Burgess Shale alone [1–3]. Phylogenetically, most of the Cambrian species, except two recently described from China [4,5], were recovered as stem-group annelids [3,4,6,7,10]. The body plan of the ancestral annelid has also been discussed in phylogenomic studies, which are largely in agreement that it was a segmented polychaete-like worm with prominent parapodia and chaetae and a head with sensory organs [11–13]. The morphologies of Cambrian stem-group annelids described to date are mostly congruent with these expectations [1–3,6–9]. Recent studies have also demonstrated that the ecology of Cambrian annelids was multifarious, with some species dwelling in tubes [4,14] or having a commensal relationship with enteropneusts [14]. Although Cambrian species have revealed many features of the first annelids, including constraints on the early evolution of the head [3], the small number of available Cambrian species and well-preserved specimens has so far limited our understanding of their early evolutionary history and ecological diversity.

Here, we describe a new Cambrian polychaete, *Ursactis comosa* gen. et sp. nov., from two geographically and stratigraphically distinct localities of the Burgess Shale Formation (British Columbia, Canada). This species expands the known disparity of polychaetes of the Burgess Shale and, more broadly, provides new insights into their ecological diversity and putative ancestral mode of growth.

## 2. Materials and methods

### 2.1. Fossil material and geological setting

**Figure 1.**
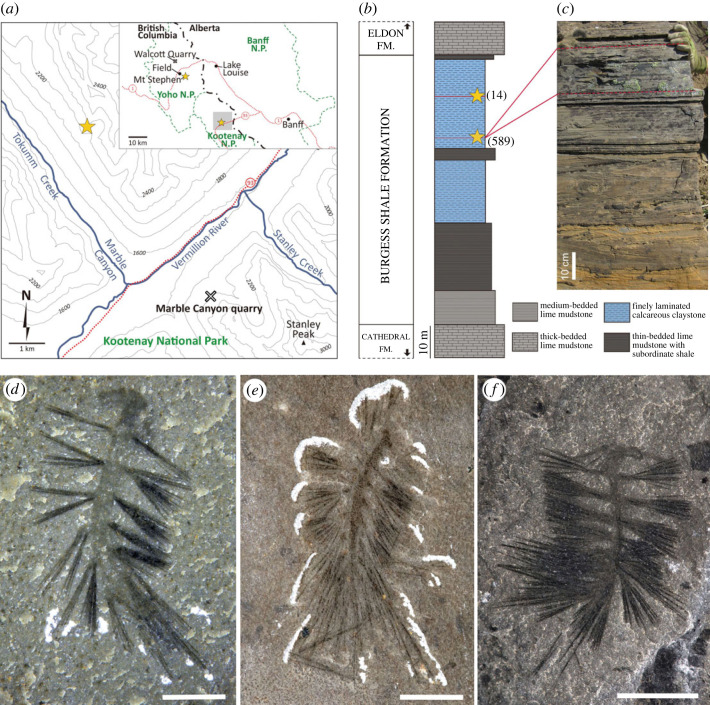
Distribution of *Ursactis*. (*a*) Maps with localities yielding *Ursactis* indicated by stars. The detailed map comes from the grey area in the general map. (*b*) Generalized stratigraphic column for the Burgess Shale Formation at the Tokumm Creek locality. The stars and the adjacent numbers represent the locations of two quarried intervals that yielded specimens of *Ursactis* and the number of specimens collected at each interval, respectively. (*c*) Outcrop photo of the lower quarried interval; the quarried interval is marked with dashed lines. (*d*–*f*) Specimens collected from the Collins Quarry on Mt. Stephen (*d*), the upper interval of the Tokumm Creek locality (*e*) and the lower interval of the Tokumm Creek locality (*f*). (*d*) *Ursactis* sp., ROMIP 66882. (*e*) *Ursactis comosa*, ROMIP 66873. (*f*) *U. comosa*, ROMIP 66874.1. Scale bars: (*d*): 1 mm; (*e*,*f*): 5 mm.

**Figure 2.**
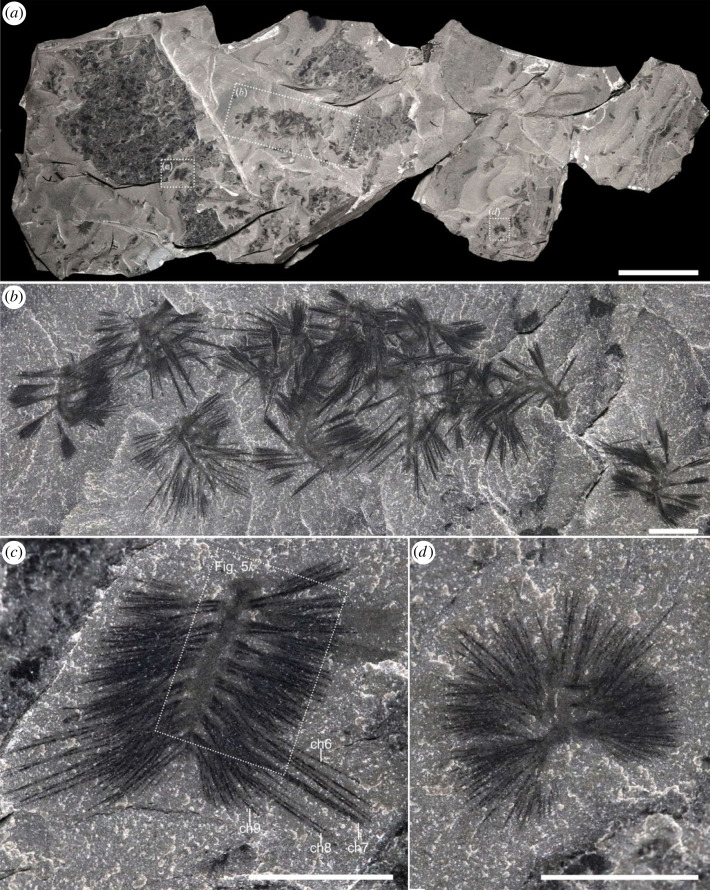
*Ursactis comosa* gen. et sp. nov. from the lower quarried interval of the Tokumm Creek locality. (*a*) Overview of one of the multiple slabs of ROMIP 66771B, preserving specimens ROMIP 66771.28–48,74,119–121. Squares with dashed lines indicate areas shown in close-up in (*b*–*d*). (*b*) ROMIP 66771.33–42. A cluster of at least 10 specimens. Counterpart to figure 3*d*. (*c*) ROMIP 66771.120. A ventrally preserved specimen showing the biramous state of parapodia, posterior longer notochaetae and the mouth. Close-up image of the head and parapodia is in figure 5*k*. (*d*) ROMIP 66771.48. An enrolled specimen. Acronym: ch, chaetae, with numbers denoting the segments, counting from the anterior, except for the peristomium. Scale bars: (*a*): 5 cm; (*b–d*): 5 mm.

**Figure 3.**
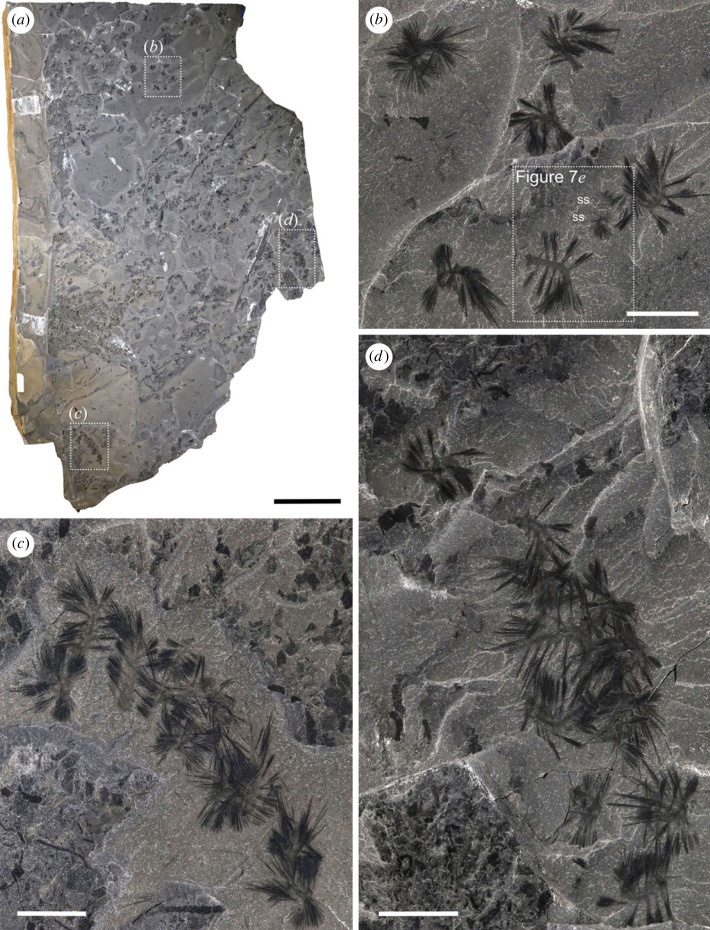
Clustered occurrence of *Ursactis comosa* gen. et sp. nov. from the lower quarried interval of the Tokumm Creek locality. ROMIP 66771A. (*a*) Overview of a large slab, which includes 70 specimens (ROMIP 66771.1–70). Squares with dashed lines indicate areas shown in close-up in (*b*–*d*). (*b*) ROMIP 66771.61–68. A cluster of eight specimens, including two small individuals (ss). Close-up of the area inside the dashed line is shown in figure 7*e*. (*c*) ROMIP 66771.2–11. A cluster of at least 10 specimens. (*d*) ROMIP66771.33–42. A cluster of at least 10 specimens. Counterpart to figure 2*b*. Acronym: ss, small specimen. Scale bars: (*a*): 10 cm; (*b*–*d*): 1 cm.

**Figure 4.**
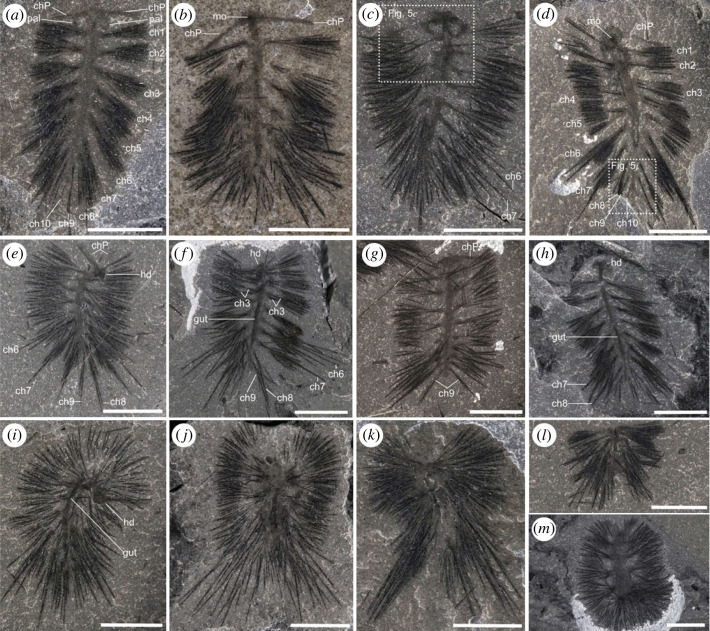
Overview of the general morphology of *Ursactis comosa* gen. et sp. nov. from the lower quarried interval of the Tokumm Creek locality. (*a*) ROMIP 66771.109. A dorsally preserved specimen exhibiting a pair of palps, peristomial neurochaetae and 10 chaetigerous segments. (*b*) ROMIP 65401.7. A ventrally preserved specimen showing the peristomial neurochaetae and the mouth. (*c*) ROMIP 66775.6. A dorsally preserved specimen showing a pair of palps, peristomial neurochaetae and longer notochaetae at the sixth and seventh segment. (*d*) ROMIP 66876.1. A ventrally preserved specimen showing the mouth, peristomial neurochaetae, 10 chaetigerous segments and the pygidium. Close-up of the posterior part of the body including the pygidium is shown in figure 5*j*. (*e*) ROMIP 66769.1. A partially bent specimen with longer notochaetae visible from the sixth to ninth segments. (*f*) ROMIP 66770.6. A dorsally preserved specimen showing the biramous state of parapodia, the gut and longer notochaetae from the sixth to ninth segments. (*g*) ROMIP 66878.1. A specimen with nine chaetigerous segments. (*h*) ROMIP 66771.101. A specimen with gut and longer notochaetae on the seventh and eighth segments. (*i*) ROMIP 65401.26. A bent specimen with gut and longer posterior notochaetae. (*j*) ROMIP 66772. A partially enrolled specimen with longer posterior notochaetae. (*k*) ROMIP 66773.10. A bent specimen with longer posterior notochaetae. (*l*) ROMIP 65401.14. An enrolled specimen. (*m*) ROMIP 66771.113. An enrolled specimen. Acronyms: ch, chaetae, with numbers denoting the segments, counting from the anterior except for the peristomium; chP: peristomial neurochaetae; hd, head; mo, mouth; pal, palp. Scale bars: 5 mm.

**Figure 5.**
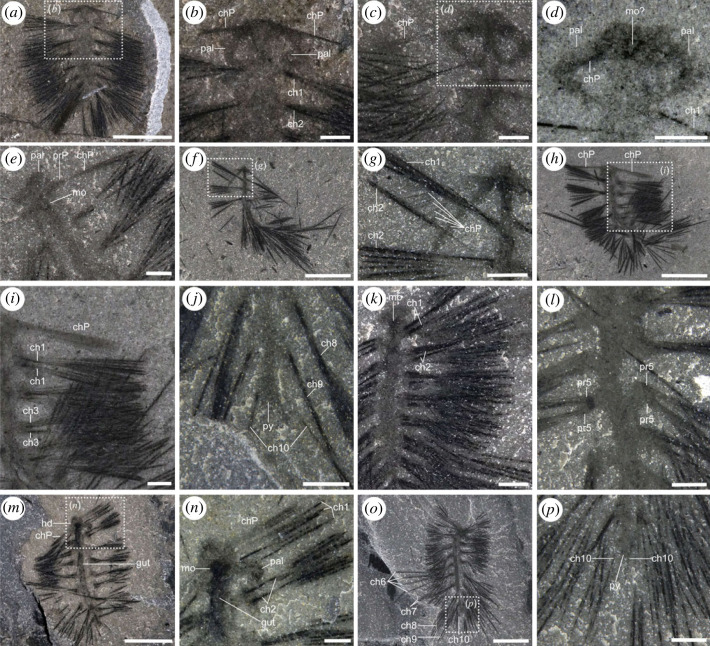
Head, parapodial, chaetal and pygidial morphology of *Ursactis comosa* gen. et sp. nov. from the lower quarried interval of the Tokumm Creek locality. (*a*,*b*) ROMIP 65401.1. (*a*) Overview of the specimen. (*b*) Close-up of the box in (*a*), showing head morphology, including palps and tightly bunched peristomial neurochaetae. (*c*,*d*) ROMIP 66775.6. (*c*) Close-up of the anterior part of the specimen boxed in figure 4*c*. (*d*) Close-up of the head, showing dark patches preserved within the head, presumably the mouth. Image taken underwater. (*e*) ROMIP 66879.3. A ventrally preserved specimen showing the mouth, peristomial neurochaetae and peristomial neuropodia. (*f*,*g*) ROMIP 66770.4. (*f*) Overview. (*g*) Close-up of the boxed area in (*f*), showing a bundle of peristomial chaetae appearing as a fan. (*h*,*i*) ROMIP 66774.1. (*h*) Overview. (*i*) Close-up of the box in (*h*), showing a bundle of peristomial neurochaetae and both noto- and neuro-chaetae. (*j*) ROMIP 66876.1 close-up of the pygidium. Overview of the specimen is shown in figure 4*d*. (*k*) ROMIP 66771.120. Overview of the specimen shown in figure 2*c*. Both bundles of noto- and neuro-chaetae are shown, indicating the biramous state of parapodia. (*l*) ROMIP 66880. A specimen showing biramous parapodia with noto- and neuro-chaetae projected towards slightly different directions. (*m*,*n*) ROMIP 66771.1. A ventrally preserved specimen showing the gut and mouth. (*m*) Overview. (*n*) Close-up of the head with the mouth. (*o*,*p*) ROMIP 66771.75. (*o*) Overview. (*p*) Close-up of the area boxed in (*o*), showing chaetae of the tenth segment and the pygidium. Acronyms: ch, chaetae; chP, peristomial neurochaetae; hd, head; mo, mouth; pal, palp; pr, parapodium; prP, peristomial neuropodium; py, pygidium; numbers following the acronyms denote segments, counting from anterior, excluding peristomium. Scale bars: (*a*,*f*,*h*,*m*,*o*): 5 mm; (*b*–*e*,*g*,*i*–*l*,*n*,*p*): 1 mm.

**Figure 6.**
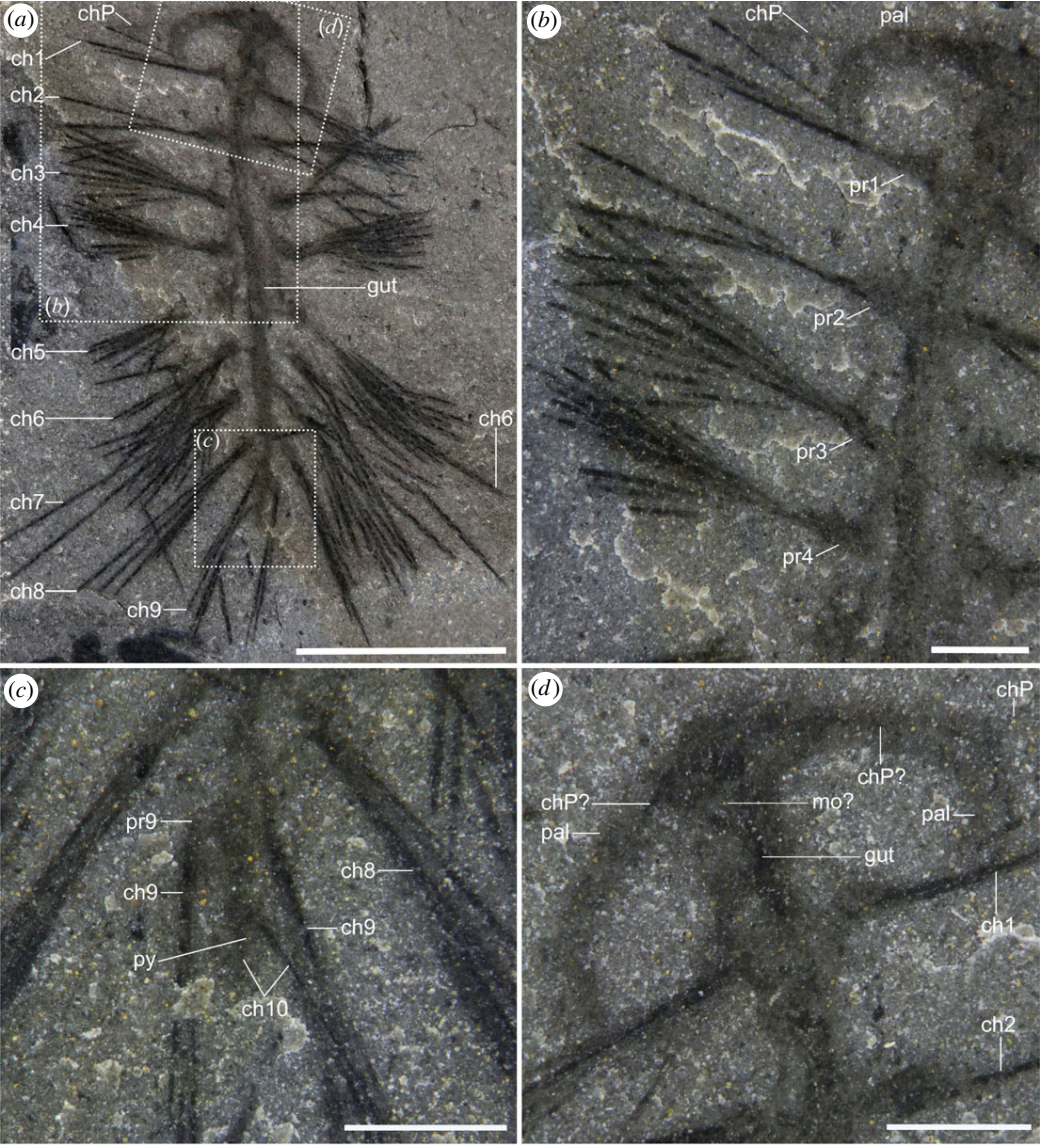
Photos of the holotype, ROMIP 66770.1, of *Ursactis comosa* gen. et sp. nov. from the lower quarried interval of the Tokumm Creek locality. The specimen has 10 chaetigerous segments and stout palps, peristomial neurochaetae, and longer notochaetae on the sixth to ninth segments. It is presumably preserved dorsally. (*a*) Composite image of part and counterpart. Squares with dashed lines indicate the area shown in (*b–d*). (*b*) Close-up of the first four segments in the part. (*c*) Close-up of segments 8–10 and the pygidium in the counterpart. (*d*) Close-up of the head in the counterpart, showing dark patches within the head. Acronyms: ch, chaetae; chP, peristomial chaetae; mo, mouth; pal, palp; pr, parapodium; py, pygidium; numbers following the acronyms denote segments, counting from anterior, excluding peristomium. Scale bars: (*a*): 5 mm; (*b*–*d*): 1 mm.

**Figure 7.**
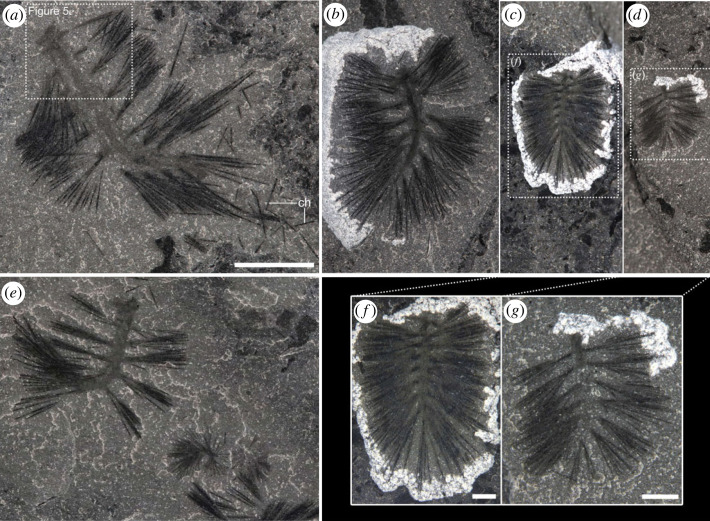
Size comparison of specimens of *Ursactis comosa* gen. et sp. nov. All the specimens were collected from the lower interval of the Tokumm Creek locality. (*a*–*e*) A series of specimens representing the range of size in *U. comosa*. The five images are all at the same scale, with a scale bar of 5 mm shown in (*a*). (*a*) ROMIP 66879.3. The largest complete specimen, approximately 15 mm long, with 10 segments. Dissociated chaetae (ch) are preserved around the specimen. A close-up of the head is shown in figure 5*e*. (*b*) ROMIP 66875. Medium-sized specimen approximately 10 mm long, 10 segments. (*c*) ROMIP 66877. The smallest specimen among those exhibiting nine segments, approximately 5 mm long. (*d*) ROMIP 66881. The smallest complete specimen, approximately 3.5 mm long, with eight segments. (*e*) ROMIP 66771.64,66,67. Three specimens, two small and one medium-sized, within a cluster, representing an occurrence of specimens of mixed body sizes within a cluster. Overall image of the cluster is shown in figure 3*b*. (*f*) Close-up of (*c*), ROMIP 66877. (*g*) Close-up of (*d*), ROMIP 66881. Acronym: ch, chaetae. Scale bars: (*a*–*e*): 5 mm; (*f*,*g*): 1 mm.

Most specimens were collected by the Royal Ontario Museum between 2018 and 2022 along Tokumm Creek in Kootenay National Park, British Columbia (figure 1*a*). The locality is approximately 6 km northwest of the Marble Canyon quarry [15–19] (figure 1*a*). At this new locality, *Ursactis* occurs in two stratigraphic intervals in the upper shale portion of the Burgess Shale Formation, approximately 13.0 and 25.8 m below the contact with the overlying Eldon Formation (figure 1*b,c*). Over 580 specimens of *Ursactis* were collected *in situ* from the lower interval within a 15 cm thick section (figures 1*f* and 2–7) and 14 specimens from the upper interval within a 40 cm thick section (figure 1*e*). Specimens occur in large and dense clusters in the lower interval (figures 2 and 3), with up to 35 specimens per cluster. Associated species in both intervals include arthropods and chordates, with abundant algae (*Dalyia* sp., *Fuxianospira* sp.) in the lower interval. Full identification of the faunal assemblages is beyond the scope of this study.

Both quarried intervals lie stratigraphically lower within the Burgess Shale than other fossil-bearing intervals so far excavated in the Marble Canyon area, including the Marble Canyon quarry, which typically occur within the upper 5 m of the Burgess Shale [18,19]. The intervals bearing *Ursactis* are the oldest fossil assemblages yet explored from the Marble Canyon area.

Both quarried intervals comprise millimetre-laminated claystones deposited below a storm wave base. Burrows are absent from intervals bearing *Ursactis*, suggesting that oxygen levels were not sufficient to promote colonization by an infauna. Like at the Marble Canyon quarry, slumps and slide dislocation features ranging from a few centimetres to over 1 m in thickness are pervasive, indicating that the depositional environment lay on a steep slope that was prone to failure [18]. These and other features of the overall regional geology suggest that the shale in the Marble Canyon area was deposited offshore from a significant break in slope formed by the Cathedral Escarpment [18,20,21]. As elsewhere in the Burgess Shale, soft-bodied fossils at the front of the escarpment were entombed by event-driven deposition of clay-dominated sediments and preserved in exquisite detail [18,22].

In addition to the specimens from Tokumm Creek, one specimen of *Ursactis* sp. (figure 1*d*), discovered in 1983, comes from the Collins Quarry on Mt. Stephen. This fossil site occurs within the lowest member of the Burgess Shale Formation, the Kicking Horse Shale Member, as defined in the type area in Yoho National Park [21,23]. It lies within the *Glossopleura* trilobite biozone [23] and is therefore older than the sites at Tokumm Creek, where *Ursactis* occurs in great abundance.

### 2.2. Fossil observation and preservation

The specimens were observed under stereomicroscopes equipped with cross-polarized filters. Some specimens partially buried under the matrix were mechanically prepared using a micro-engraver equipped with a carbide bit. Digital photographs of the dry and wet specimens were taken under direct or cross-polarized light, and all photographs in this paper were taken with cross-polarized light. Morphological measurements were taken using photographic images of complete and nearly complete specimens using ImageJ 1.51n [24].

As is typical of Burgess Shale fossils, specimens of *Ursactis* are preserved as carbonaceous and aluminosilicate compression films [25]. The chaetae appear darker than the rest of the body, presumably due to a higher carbon concentration resulting from their originally chitinous composition. Darker areas also occur where the chaetae bundles are tightly packed, for example, as they emerge from the parapodia (e.g. figure 5*k*). The gut and internal areas within the head also tend to preserve as a darker film (e.g. figure 6*a*,*d*). The outlines of the body and parapodia are often faintly preserved. Most specimens appear complete or nearly complete, but the head and posterior ends are often concealed by the matrix or chaetae bundles. In addition, under 10% of the specimens are preserved lying straight and flat (figures 2*c*, 4*a*–*d*,*g*,*h*, 6*a*, and 7*f*). Instead, most specimens are buried at various angles or partially enrolled (e.g. figures 2*d*, 3*b*, 4*e*,*f*,*i*–*m*, and 5*f*,*h*,*o*), which also explain why the head and posterior end are also most commonly concealed. Dissociated chaetae preserved on the same slabs with complete specimens (e.g. figure 7*a*) suggest advanced pre-burial decay in some specimens [26]. Considering that there is no evidence of sorting or current orientation of any specimens, including those of other species, large clusters of individual specimens buried on top of each other (e.g. figures 2*b* and 3*c*,*d*) suggest that transportation during burial was probably minimal and that the community was buried mainly *in situ*. Curled-up individuals might indicate post-mortem artefacts (i.e. muscle contraction) or potential escape behaviour of live specimens, similar to what has been reported for other Burgess Shale species, such as *Pikaia gracilens* [27]. The fact that nearly 90% of specimens are preserved curled to some degree supports a mass mortality event during burial.

### 2.3. Phylogenetic analysis

We conducted a Bayesian phylogenetic analysis using a character matrix of Chen *et al*. [4], which was originally compiled by Parry *et al*. [10] and subsequently updated with additions and modifications of taxa and characters [3,28–30]. *Ursactis* was added to the matrix, and some of the character codings for Cambrian annelids were modified (see electronic supplementary material for details). Our character matrix comprises 144 taxa and 364 characters (electronic supplementary material). Bayesian analysis was performed using MrBayes 3.2.7a [31] using the Mkv model with the rate variation set to a gamma distribution (electronic supplementary material). The program was instructed to stop the tree exploration when the average standard deviation of split frequencies reached 0.01 to confirm the convergence of the resulting trees, and the analysis ran for 22 465 000 generations. Sampling was done every 1000 generations, and the first 25% of trees were discarded as burn-in. A consensus tree was obtained by the 50% majority rule. Stationarities of the runs were confirmed by checking the effective sample size scores of all the parameters higher than 200 using Tracer 1.7.1 [32]. Tree visualizations were done using FigTree 1.4.4 [33], and the tree was rooted at *Amphiporus* (Nemertea).

## 3. Fossil descriptions

### 3.1. Systematic palaeontology

Phylum. Annelida Lamarck, 1809.

*Ursactis comosa* gen. et sp. nov.

LSID urn:lsid:zoobank.org:act:9523FD71-3A28-4FE9-A46F-EE765CE67983

LSID urn:lsid:zoobank.org:act:0F471000-DFA6-4475-AC9B-91D0E9C1C71A

### 3.2. Etymology

*Ursa* for the constellation Ursa Major, and *actis*, a Greek word for a ray, referring to the species' starry appearance and the clustered occurrence of fossils; *comos*, a Latin word meaning ‘having long hair,’ for its long capillary chaetae, in particular some notochaetae on segments six to nine.

### 3.3. Type material

Holotype: ROMIP66770.1 (figure 6).

Paratypes: ROMIP65401.1 (figure 5*a*,*b*), ROMIP65401.7 (figure 4*b*), ROMIP65401.14 (figure 4*l*), ROMIP65401.26 (figure 4*i*), ROMIP66769.1 (figure 4*e*), ROMIP66770.4 (figure 5*f*,*g*), ROMIP66770.6 (figure 4*f*), ROMIP66771.1 (figures 3*a* and 5*m*,*n*), ROMIP66771.2–11 (figure 3*a*,*c*), ROMIP66771.28-32,43-47 (figures 2*a* and 3*a*), ROMIP66771.33-42 (figures 2*a*,*b* and 3*a*,*d*), ROMIP66771.48 (figures 2*a*,*d* and 3*a*), ROMIP66771.61-63,65,68 (figure 3*a*,*b*), ROMIP66771.64,66,67 (figures 3*a*,*b* and 7*e*), ROMIP66771.75 (figure 5*o*,*p*), ROMIP66771.101 (figure 4*h*), ROMIP66771.109 (figure 4*a*), ROMIP66771.113 (figure 4*m*), ROMIP66771.120 (figures 2*a*,*c* and 5*k*), ROMIP66772 (figure 4*j*), ROMIP66773.10 (figure 4*k*), ROMIP66774.1 (figure 5*h*,*i*), ROMIP66775.6 (figures 4*c* and 5*c*,*d*), ROMIP66873 (figure 1*e*), ROMIP 66874.1 (figure 1*f*), ROMIP66875 (figure 7*b*), ROMIP66876.1 (figures 4*d* and 5*j*), ROMIP66877 (figure 7*c*,*f*), ROMIP66878.1 (figure 4*g*), ROMIP66879.3 (figures 5*e* and 7*a*), ROMIP66880 (figure 5*l*), ROMIP66881 (figure 7*d*,*g*), ROMIP66882 (figure 1*d*) and 537 unfigured specimens (see electronic supplementary material for the full list of specimens).

### 3.4. Locality and stratigraphy

One specimen of *Ursactis* sp. (figure 1*d*) comes from the Collins Quarry on Mt. Stephen, Yoho National Park, Kicking Horse Shale Member [21,23]. All the specimens of *U. comosa* come from two stratigraphic intervals within the upper part of the Burgess Shale Formation, Cambrian (Miaolingian Series, Wuliuan Stage), *Ehmaniella* biozone, at the Tokumm Creek locality of the Marble Canyon area, northern Kootenay National Park, British Columbia, Canada [15,18].

### 3.5. Diagnosis for genus and species

Polychaete worm possessing maximum 10 chaetigers, excluding the peristomium, with biramous parapodia yielding simple capillary chaetae. Approximately 8–12 chaetae on each neuropodium and notopodium, and among chaetigers one to eight. The last two chaetigers have approximately five and two chaetae, respectively. Up to five notochaetae on chaetigers six to nine, double the length of other chaetae on the same chaetigers.

### 3.6. Description

**Figure 8.**
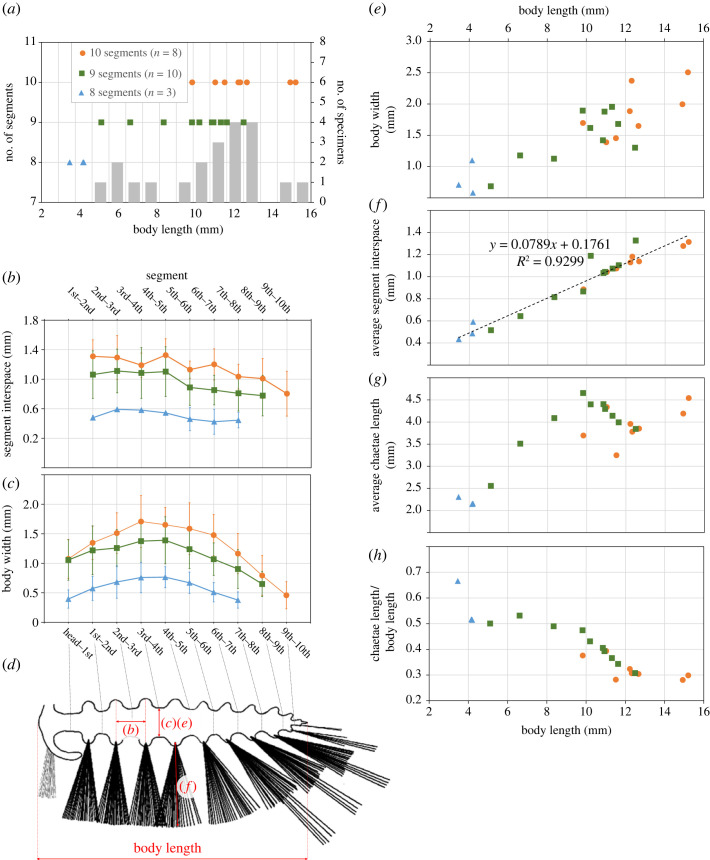
Results of morphometric measurements in *Ursactis comosa* gen. et sp. nov. Only complete or nearly complete specimens showing the whole-body trunk was used for measurements; 21 specimens in total. (*a*) Distribution of the number of segments shown in the specimens (dots) and the number of measured specimens (bars) relative to body length of specimens. (*b*) Segment interspace from the first to the last segment, representing the length of each segment, averaged among specimens with the same number of segments. Error bars indicate standard deviation. (*c*) Body width between segments from the head to the last segments, averaged among specimens with the same number of segments. Error bars indicate standard deviation. (*d*) Diagram of the body of *Ursactis* with arrows indicating the parts measured for body length (*a*,*e*–*h*), segment interspace (*b*), body width (*c*) and chaetae length (*f*). (*e*) Relationship between body length and width (maximum body width of each specimen). Each dot represents one specimen. (*f*) Relationship between body length and segment interspace averaged among all segments. A significant positive correlation between body length and segment interspace (*r* = 0.964, *p* < 0.001) was observed. (*g*) Relationship between body length and the average length of the five longest chaetae at the anterior-most five segments (excluding the peristomium). (*h*) Relationship between body length and the average length of the five longest chaetae on the five anterior segments relative to body length.

**Figure 9.**
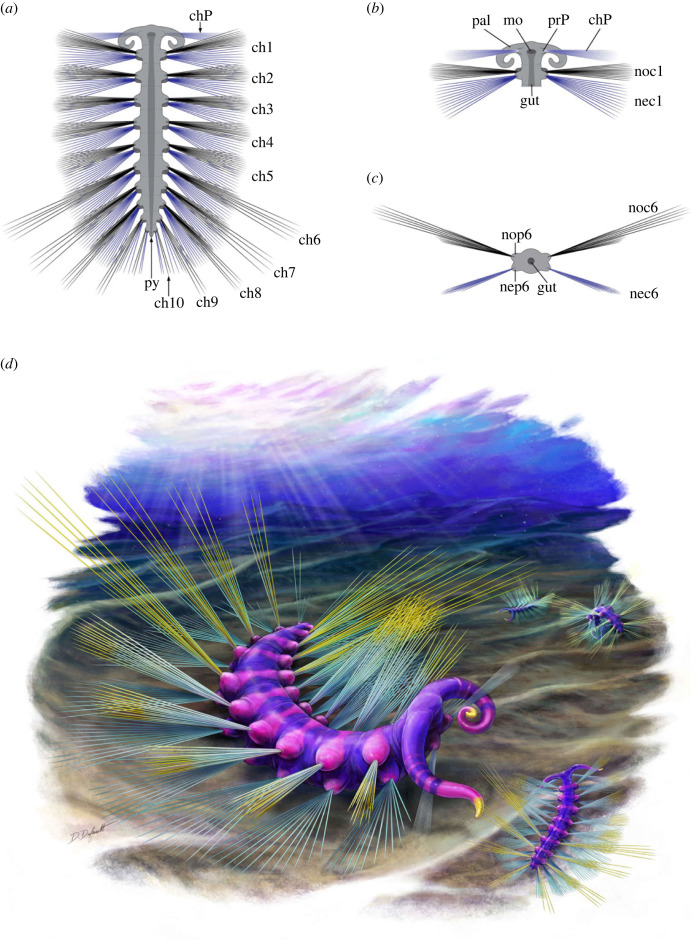
Technical drawing and life reconstruction of *Ursactis comosa* gen. et sp. nov. (*a*–*c*) Technical drawing. Black and blue chaetae indicate noto- and neuro-chaetae, respectively. (*a*) Overview of the body in a dorsal view. (*b*) Ventral view of the head with palps, peristomial neuropodia and neurochaetae, and mouth. (*c*) Cross-section of the sixth segment. (*d*) Life reconstruction of *Ursactis*. All drawings by Danielle Dufault © Royal Ontario Museum. Acronyms: ch, chaetae; chP, peristomial neurochaetae; mo, mouth; nec, neurochaetae; nep, neuropodium; noc, notochaetae; nop, notopodium; pal, palp; prP, peristomial neuropodium; py, pygidium; numbers denote segments, counting from anterior, except for the peristomium.

The body is slender in overall appearance, ranging between *ca* 3 and 15 mm in length (excluding the chaetae and palps) and *ca* 0.6 and 2.5 mm in width (figure 8*e*). The body bears 8–10 chaetigers, excluding the peristomium (figures 6 and 9*a*), with the smaller specimens bearing fewer chaetigers (eight or nine) in general (figure 8*a*). The length of each chaetiger is largely constant from the first to around the fifth chaetiger and then slightly shortens towards the posterior (figure 8*b*). The trunk is widest in its middle, around the third and fourth chaetigers, counting from the anterior (excluding the peristomium), and tapers gradually towards the posterior (figure 8*c*). The pygidium is small (figures 5*j*,*p* and 6*c*) and often concealed. There is no trace of pygidial cirri (figures 5*j*,*p* and 6*c*).

The head is oval (approx. twice as wide as long) and bears a pair of stout, elongated palps (e.g. figure 6*d*). The head is slightly wider than the body and represents *ca* 10% of the total body length (excluding chaetae and palps) (e.g. figure 6*a*). The head has a round front and is gently convex towards the anterior (e.g. figure 6*d*). No median or lateral antenna was observed. The palps are smooth and robust, and their length is approximately a fifth of the total body length (e.g. figure 6*a*,*d*). The palps are attached laterally to the head and are usually arcuate and partially enrolled (e.g. figures 5*b* and 6*d*). Palp width at the base can be close to the length of the head, and palps taper gradually towards the tip (e.g. figure 6*d*); in the holotype specimen *ca* 13 mm long (figure 6), the palps are *ca* 2 mm long, *ca* 0.6 mm wide at the base and *ca* 0.4 mm wide at the middle point. A nearly bilaterally symmetrical, round to heart-shaped spot seemingly connected to the gut is preserved at the centre of several heads (e.g. figure 5*n*). Occupying *ca* 15% of the head, these dark spots are interpreted as traces of the mouth. As in the Burgess Shale polychaetes, *Canadia* [3,29,34], *Burgessochaeta* [2,3], and *Kootenayscolex* [3], a pair of thin peristomial chaetal bundles projects laterally from the head (e.g. figures 4*a* and 5*b*,*h*). The bases of these bundles are located slightly anterior to and on either side of the mouth (e.g. figures 4*a* and 5*e*). These peristomial chaetae appear as a fan of thin filaments (figure 5*g*) or within a tightly bunched bundle (e.g. figure 5*b*,*e*). The peristomial chaetae are slightly shorter than the chaetae on the bodies (e.g. figure 5*i*) and *ca* 3 mm long in the largest specimens, including the holotype. They are also extremely thin, at most *ca* 0.01 mm (figure 5*g*) and significantly thinner than the chaetae belonging to the body. The exact number of peristomial chaetae per chaetal bundle is unclear but may reach up to 20 (figure 5*g*). The peristomial chaetae originate from a bulbous parapodium, which is smaller in size compared with the parapodia along the body (figures 5*e* and 7*a*). Peristomial chaetae and parapodia of *Ursactis* are typically clearly preserved in ventral specimens with a mouth (figures 4*b*, 5*e*,*n*, and 7*a*), but less often with complete palps, leading to the interpretation that the peristomial chaetae/parapodia are uniramous and are ventral neurochaetae/neuropodia (figure 9*b*).

Based on the presence of two distinct bundles of chaetae originating from the same area (figure 5*i*,*k*,*l*,*n*), parapodia along the body are interpreted to be biramous. The parapodia are bulbous (e.g. figures 5*l* and 6*b*). The size and shape of notopodia and neuropodia appear nearly identical (figure 5*l*), although this is difficult to establish with certainty owing to the small size of these features. Each noto- and neuro-podium possesses approximately 8–12 capillary chaetae. This number seems largely constant between noto- and neuro-podia as well as among the chaetigers until the eighth chaetiger. We observe a maximum of five and two chaetae on each side of the body in the ninth and tenth chaetiger, respectively (figure 6*c*), but because of potential overlap between chaetae and relatively poor preservation, these numbers could be higher. The chaetae project roughly laterally in the anterior half of the body and gradually become more projected towards the back in the posterior half (e.g. figures 4*a* and 6*a*). However, chaetae are often directed anteriorly or posteriorly to various extents within a specimen (e.g. figures 5*f*,*h*,*m* and 7*a*), indicating that the orientations of parapodia/chaetae were quite flexible in life. There is no trace of aciculae, parapodial cirri or branchiae.

In medium to large specimens *ca* 8–15 mm long, including the holotype, the length of chaetae is approximately 4 mm, and this length is largely constant between noto- and neuro-chaetae as well as across the body (e.g. figures 5*i*,*k* and 6*a*), except for the chaetae on the ninth and tenth chaetigers and the prominent longer chaetae described below. Chaetae on chaetigers 9 and 10 are probably shorter than other chaetae on the body (e.g. figure 6*a*). The width of the chaetae, with the exception of the peristomial neurochaetae, is *ca* 0.05 to 0.08 mm and is largely constant across the body until the eighth chaetiger, regardless of their length. The chaetae in chaetigers 9 and 10 appear slightly thinner than the rest (figure 6*c*). Chaetigers six to nine bear chaetae significantly longer than other chaetae on the same chaetiger. Chaetigers six to eight bear up to five longer chaetae on each side out of the 12 chaetae in a bundle (e.g. figures 4*e*,*f* and 6*a*). In medium to large specimens, the lengths of the longest chaetae are *ca* 5–9 mm (e.g. figures 4*e*,*f* and 6*a*). Chaetiger nine bears up to three longer chaetae on each side of the body within a bundle of approximately five chaetae in total, though the longest chaetae are slightly shorter than the longest chaetae of the anterior chaetigers six to eight (figures 4*e*,*f* and 6*a*). We interpret the ‘longer’ chaetae on chaetigers six to nine as notochaetae (figure 9*c*) based on the fact that, in ventral specimens, these longer chaetae are usually not preserved on the same bedding plane and are often partially covered by shorter chaetae belonging to neuropodia (figure 2*c*).

The alimentary canal is often preserved as a straight tube running along the median dorsoventral axis of the body trunk from the mouth to the posterior part of the body, although the outline of the canal fades away gradually towards the posterior (e.g. figures 5*m* and 6*a*). It is *ca* 0.3–0.6 mm wide in medium to large specimens, around one-third of the body width (e.g. figures 5*m* and 6*a*,*b*).

## 4. Results and discussion

### 4.1. Comparative morphology

The capillary chaetae of *Ursactis* are particularly similar in morphology to *Phragmochaeta* [8] and *Kootenayscolex* [3]: they are thin and long relative to their body size, and the chaetal fascicles fan out along the body. The notochaetae of Cambrian polychaetes are considered to be primarily defensive in nature [34,35]. The longer posterior notochaetae, which are unique to this species, would have presumably enhanced the defensive function, especially in enrolled specimens. In such specimens, the head becomes effectively concealed under the posterior part of its body, while the longer notochaetae project outward protecting the posterior part of the body and pygidium. In extant benthic annelids, shorter capillary chaetae may be used for stabilizing the body inside the burrows or tubes in which they dwell [36]. However, the notochaetae of *Ursactis* seem less likely to be used for such a purpose because they have no structures that can be used for anchoring (e.g. hooks) nor aciculae that would make the parapodia or chaetae more rigid. In addition, the longer chaetae are only seen in the posterior segments, making anchoring as the main function questionable. The ventral neurochaetae, which are all similar in size and shape, are interpreted to be mainly used for locomotion in crawling along the seafloor.

The paired palps and the peristomial chaetae on the head are features that *Ursactis* shares with *Canadia* [3,34], *Burgessochaeta* [2,3] and *Kootenayscolex* [3], although the peristomial chaetae are seemingly borne by biramous parapodia in *Canadia* [29]. Paired palps are often observed in other Cambrian annelids as well [2,4,9,34], and their presence is recognized as an ancestral state of the Annelida in phylogenetic studies [12]. The palps of *Ursactis* appear stouter than the elongated palps of other Cambrian polychaetes, and the attachment of the palps is more posterior than in other worms from the Burgess Shale with elongated palps, such as *Canadia*, *Burgessochaeta* and *Kootenayscolex*, but more similar in position to those in *Guanshanchaeta* from China [9]. The palps of Cambrian stem-group polychaetes are suggested to have had sensory and feeding functions [29], an explanation which we tentatively extend to those in *Ursactis*.

Finally, our study demonstrates that the presence of peristomial chaetae is relatively common in Cambrian stem-group annelids. The heads of four taxa, *Canadia* [34], *Burgessochaeta* [2,3], *Kootenayscolex* [3] and *Ursactis,* are interpreted to possess peristomial chaetae, suggesting that the ancestral annelid possessed peristomial chaetae as well (the ‘chaetigerous mouth hypothesis' in Nanglu & Caron [3] and its references).

### 4.2. Palaeoecology

The preservation of several large clusters of specimens of *Ursactis* suggests a gregarious habit. Extant polychaetes such as syllids and nereidids are known to exhibit reproductive swarming behaviour, in which adult worms aggregate for mating and spawning [37,38]. Most of the specimens in the preserved clusters seem to be homogeneous in size, although some specimens are much smaller (figure 7*e*) and may represent juveniles or young adults. While these smaller individuals may be unrelated to the clusters, they are unlikely to be involved in reproductive swarming. Since there is no evidence of sexual dimorphism or epitokes, reproductive swarming is difficult to support. Other possibilities for swarming behaviour could include feeding or putative protection from predators. If the swarming were related to feeding, it would indicate that the seafloor was a rich source of nutrients, allowing large colonies of this species to survive. Swarming to escape predation is more speculative. Although we are not aware of any modern examples of polychaetes engaging in such behaviour, other modern species, including arthropods and vertebrates, have evolved such behaviour in response to predation pressure [39].

The feeding mode of *Ursactis* would most likely involve its long palps to sweep food towards its mouth, where the absence of tentacles may also discount a suspension-feeding lifestyle. *Ursactis* does not seem to have been a raptorial predator because there is no trace of an apparatus for catching prey (e.g. jaws or a muscular proboscis). Also, its long and thin capillary chaetae without aciculae would not be suitable for the fast and powerful movement needed for hunting.

The combination of morphological characters and the exceptional preservation of large numbers of specimens buried together suggest that *Ursactis* was an epibenthic surface deposit feeder (figure 9*d*). It might have used its dorsal notochaetae for protection against predators and its ventral neurochaetae for locomotion on the seafloor. Its high density indirectly supports the presence of a rich food source along the seafloor, which was already indicated by large concentrations of the radiodont *Cambroraster* with specialized rake-like appendages [16].

### 4.3. Phylogenetic results

**Figure 10.**
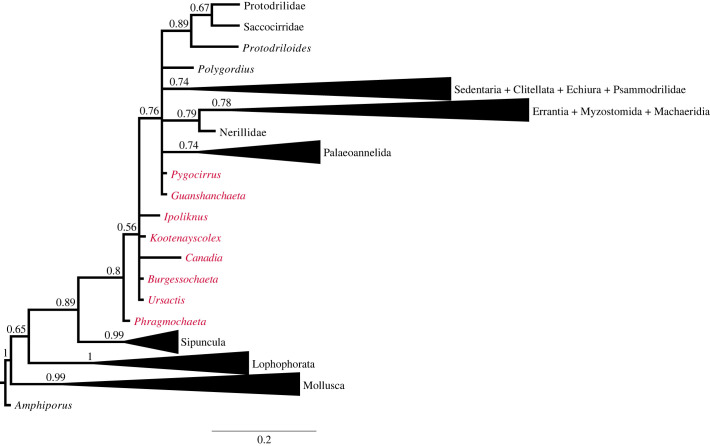
Fifty per cent majority-rule consensus tree derived from Bayesian analysis, showing the phylogenetic position of *Ursactis*. The numbers on nodes are posterior probabilities. Taxa in red indicate Cambrian annelids. The branch length represents expected changes per site. See the electronic supplementary material for the full result.

Our Bayesian phylogenetic trees (figure 10; electronic supplementary material) were largely consistent with previous studies, showing poorly resolved placement of most Cambrian polychaetes [3,4,10,29,30]. The trees situated *Ursactis* as a stem-group annelid and in a polytomy with other Cambrian annelids. The phylogenetic relationships among Cambrian annelids and other extinct/extant annelids, as well as those among outgroups (Nemertea, Phoronida and Brachiopoda), extinct/extant molluscs, sipunculans and the total annelid clade, did not show any conflict with the results of unconstrained phylogenetic analyses in Chen *et al.* [4].

### 4.4. Evolution of chaetae diversity and body tagmatization

The characteristic long dorsal chaetae of *Ursactis* have implications for the evolution of chaeta diversity within an individual and for body tagmatization in annelids. Morphological differences between dorsal notochaetae and ventral neurochaetae within individuals have been reported in some Cambrian annelids [2,6] and are most prominent in *Canadia* [2]. Those dorsoventral differences in morphology are constant across segments, except for a reduction in overall chaeta size anteriorly and posteriorly [2,6]. A possible significant anteroposterior change in chaeta size was documented in *Phragmochaeta*, whose posterior chaetae were approximately 1.5 times longer than those of its anterior segments [8]. Although the quality of preservation of the published *Phragmochaeta* material appears relatively poor, this chaetal arrangement may be similar in *Ursactis*.

Modern annelids exhibit highly diverse chaetal morphology and orientation within individuals. The most extreme cases include body tagmatization, which involves a change in the arrangement of chaetal components [40]. For example, some spionids and capitellids possess hooked chaetae only in posterior segments, and the hooked chaetae are borne together with capillary chaetae in the same chaetal bundles [40–42]. Chaetae of *Ursactis* are all capillary chaetae, and there is no difference in chaeta type. However, the occurrence of remarkably longer chaetae on specific segments suggests that body tagmatization, albeit much simpler than in extant polychaetes, already existed in the Cambrian.

### 4.5. Mode of growth in Cambrian polychaetes

The small number of body segments in *Ursactis*—a maximum of 10—may be a key to understanding the evolution of segment development in annelids. The number of segments in extant polychaetes is usually several tens to a few hundred [43]. Even polychaetes smaller than *Ursactis* have approximately the same number or more segments; for example, Nerillidae rarely exceed 2 mm in length but have seven to nine segments [40], and *Fabriciola minuta*, the smallest representative of the Sabellidae, at 0.85 mm when mature, has 11 segments [40]. The small number of segments in *Ursactis* is also unique among Cambrian stem annelids. The maximum number of segments inferred in the Burgess Shale taxa is 34 in *Peronochaeta* [2] and *ca* 20–25 in others [2,3]. Most of the stem annelids from other Cambrian localities also possess *ca* 20 segments [6,8,9], with a maximum of *ca* 50 in *Ipoliknus* [6] and a minimum of 14 in *Pygocirrus* [7]. It is worth mentioning that these counts are generally underestimates of the true total number of segments that these species would have had in life. In polychaetes, the posterior end of the body is known to be more prone to decay [44,45], and the posterior sections of fossilized specimens are rarely preserved or concealed. This makes the small number of segments in *Ursactis* even more remarkable.

The number of segments in *Ursactis* varies very little and is probably finite, which is not typical of modern polychaetes. Most modern polychaetes produce a few to a few dozen initial segments during the larval stage, and then continuously add several dozen to a few hundred more segments at the segment addition zone (SAZ) during the adult phase [43,46]. By contrast, the number of segments in *Ursactis* is highly constrained between 8 and 10, although smaller specimens of *Ursactis* have fewer segments (eight or nine) (figure 8*a*). While several modern polychaetes stop adding segments at the SAZ at some point, for example, Nerillidae and some Sabellidae [40,47], they produce more segments relative to their body size than *Ursactis* does. For instance, the number of segments in *Branchiomma bairdi* (Sabellidae) reaches a plateau at approximately 70 when it gets to *ca* 28 mm long [47].

Our data further suggest that the growth of *Ursactis* during and after its juvenile stage is primarily accomplished by an increase in size of each of the segments rather than through the generation of new segments. The distance between the parapodia of adjacent segments (as a proxy for the length of each segment), averaged within an individual, showed a significant positive correlation with body length (*r* = 0.964, *p* < 0.001) (figure 8*f*). The overlap of body sizes between specimens having 9 and 10 segments (figure 8*a*) could be in part due to the tenth segment not being preserved or visible in some specimens. However, the significant correlation between body size and interspaces between segments regardless of the number of segments (figure 8*f*) shows that this potential bias is not significant, since the last segment is also the shortest (figure 8*b*). The spread of sizes of individuals with 10 segments (figure 8*a*), and the absence of evidence of specimens with 11 or more segments, suggest that the number of segments stabilized beyond that point of development.

Conversely, chaetal growth appears to have slowed in larger specimens. The average length of chaetae increased from the smallest specimen at *ca* 3.5 mm to larger specimens at *ca* 10 mm long but did not show as dramatic an increase in the larger individuals (figure 8*g*). Overall, this trend led *Ursactis* to have shorter chaetae relative to body length in the larger specimens (figure 8*h*).

The growth pattern observed in *Ursactis* has implications for the early evolution of annelid development. As discussed above, the limited generation of segments exhibited in *Ursactis* is different from that in most extant polychaetes, in which segment numbers continue to increase after maturity. Due to the limited amount of information about late developmental phases in extinct annelids, in particular, growth through segment addition in adult specimens, and owing to the limited availability of fossils, we cannot conclude whether the developmental mode seen in *Ursactis* is ancestral or derived in annelid evolution. In addition, *Ursactis* falls in a polytomy with other stem-group annelids in our phylogeny (figure 10). Depending on whether it is ancestral or derived, two hypotheses can be proposed: (i) the addition of a defined number of segments exhibited in *Ursactis* is the ancestral state of annelids, and annelids evolved to continue generating segments after maturity in the base of crown-group annelids, with this trait being secondarily lost in some extant lineages; (ii) the mode of development of *Ursactis* is apomorphic in stem annelids, and, as one can predict from the fact that continuous segment addition is the norm in modern polychaetes, ancestral annelids produced segments throughout their lifetimes, and this trait was then carried on to the majority of the modern polychaete taxa with a few lineages independently evolving to terminate segment addition during their growth, as seen in *Ursactis*.

## 5. Conclusion

The known disparity and ecological diversity of Cambrian annelids have expanded in recent years thanks to the discovery of new species, including tube-dwelling forms [4,14] and forms living in symbiosis with other taxa [14]. The clustering behaviour of *Ursactis* also implies that some polychaetes probably occupied substantial space and resources in some areas, suggesting a greater ecological role in local ecosystems.

The morphology of *Ursactis* may provide important clues to understanding the developmental patterns of the ancestral annelid. First, the occurrence of longer chaetae on specific segments suggests an early example of simple body tagmatization in annelids. Second, the surprisingly small number of segments and minor variation in segment number in this new polychaete implies that the addition of segments was terminated earlier in ontogeny than in modern polychaetes. Continued study of the modes of growth in fossilized annelids and of post-larval development of extant polychaetes would provide important clues to understanding the evolution of developmental patterns in the Annelida.

## Data Availability

The data are provided in the electronic supplementary material [48].
